# Supplementary material for: The astrocytic ensemble acts as a multiday trace to stabilize memory
Source: Nature. 2025 Oct 15;648(8092):146–56. doi: 10.1038/s41586-025-09619-2 (PMC12675280; doi:10.1038/s41586-025-09619-2)
Supplement: Supplementary file 2 — Reporting Summary [file 41586_2025_9619_MOESM2_ESM.pdf]

nature portfolio

Corresponding author(s): Jun Nagai

Last updated by author(s): Aug 30, 2025

Reporting Summary

Nature Portfolio wishes to improve the reproducibility of the work that we publish. This form provides structure for consistency and transparency in reporting. For further information on Nature Portfolio policies, see our Editorial Policies and the Editorial Policy Checklist.

Statistics

For all statistical analyses, confirm that the following items are present in the figure legend, table legend, main text, or Methods section.

n/a	Confirmed	
☐	☒	The exact sample size (*n*) for each experimental group/condition, given as a discrete number and unit of measurement
☐	☒	A statement on whether measurements were taken from distinct samples or whether the same sample was measured repeatedly
☐	☒	The statistical test(s) used AND whether they are one- or two-sided<br>*Only common tests should be described solely by name; describe more complex techniques in the Methods section.*
☐	☒	A description of all covariates tested
☐	☒	A description of any assumptions or corrections, such as tests of normality and adjustment for multiple comparisons
☐	☒	A full description of the statistical parameters including central tendency (e.g. means) or other basic estimates (e.g. regression coefficient) AND variation (e.g. standard deviation) or associated estimates of uncertainty (e.g. confidence intervals)
☐	☒	For null hypothesis testing, the test statistic (e.g. *F*, *t*, *r*) with confidence intervals, effect sizes, degrees of freedom and *P* value noted<br>*Give P values as exact values whenever suitable.*
☒	☐	For Bayesian analysis, information on the choice of priors and Markov chain Monte Carlo settings
☒	☐	For hierarchical and complex designs, identification of the appropriate level for tests and full reporting of outcomes
☐	☒	Estimates of effect sizes (e.g. Cohen's *d*, Pearson's *r*), indicating how they were calculated

Our web collection on statistics for biologists contains articles on many of the points above.

Software and code

Policy information about availability of computer code

Data collection	QuantStudio (Applied Biosystems, 12K), CytExpert (Beckman Coulter, version 1.0.3), FlowJo (Becton Dickinson, version 10.9.0), Fluoview (FV3000, Olympus). Freezing was automatically measured throughout the testing trial by the TimeFZ4 software (version 2021_7_29, Ohara).
Data analysis	Softwares: NeuroInfo (MBL Bioscience, BNS-200), ANTs (Stnava, https://stnava.github.io/ANTs/), JupyterLab (3.6.7, Project Jupyter, https://jupyterlab.readthedocs.io/en/latest/), Python (v3.00, Python Software Foundation), Numpy (1.26.4, https://www.nature.com/articles/s41586-020-2649-2), Matplotlib (3.8.0, https://ieeexplore.ieee.org/document/4160265/authors#authors), Pandas (2.1.4, NumFOCUS, Inc., https://pandas.pydata.org/), Scipy (1.11.4, https://www.nature.com/articles/s41592-019-0686-2) , sklearn (1.2.2, RRID:SCR_002577), ImageJ (NIH), GraphPad Prism (10.1.2, GraphPad), RStudio (2023.12.1+402, Posit Software, PBC), R4.3.2 (R Core Team, RRID:SCR_001905; https://www.R-project.org/).<br>Codes: https://github.com/BrainImageAnalysis/Astrocyte3DMapping/, https://github.com/Jun-Nagai-Lab/Dewa-et-al/

For manuscripts utilizing custom algorithms or software that are central to the research but not yet described in published literature, software must be made available to editors and reviewers. We strongly encourage code deposition in a community repository (e.g. GitHub). See the Nature Portfolio guidelines for submitting code & software for further information.

Data

Policy information about availability of data

All manuscripts must include a data availability statement. This statement should provide the following information, where applicable:

- Accession codes, unique identifiers, or web links for publicly available datasets
- A description of any restrictions on data availability
- For clinical datasets or third party data, please ensure that the statement adheres to our policy

All the single-cell transcriptomics data are available at Gene Expression Omnibus with accession identifier GSE272414. For the scRNA-seq analysis, we used the mouse reference genome mm10 (GENCODE vM23/Ensembl 98), downloaded from the 10x Genomics reference data site https://cf.10xgenomics.com/supp/cell-exp/refdata-gex-mm10-2020-A.tar.gz. All raw replicate data values used to generate the figures and the associated statistical tests are provided in Supplementary Table 1. Fos-mNG astrocyte counts in 677 brain regions in NoFC, FC, NoFR and FR groups are provided in Supplementary Table 2. All raw data supporting the findings of this study are available at RIKEN CBS Data Sharing Platform https://neurodata.riken.jp/id/20250831-001 (DOI: 10.60178/cbs.20250831-001).

Research involving human participants, their data, or biological material

Policy information about studies with human participants or human data. See also policy information about sex, gender (identity/presentation), and sexual orientation and race, ethnicity and racism.

Reporting on sex and gender	n/a
Reporting on race, ethnicity, or other socially relevant groupings	n/a
Population characteristics	n/a
Recruitment	n/a
Ethics oversight	n/a

Note that full information on the approval of the study protocol must also be provided in the manuscript.

Field-specific reporting

Please select the one below that is the best fit for your research. If you are not sure, read the appropriate sections before making your selection.

☒ Life sciences ☐ Behavioural & social sciences ☐ Ecological, evolutionary & environmental sciences

For a reference copy of the document with all sections, see nature.com/documents/nr-reporting-summary-flat.pdf

Life sciences study design

All studies must disclose on these points even when the disclosure is negative.

Sample size	Sample and group sizes were determined based on data from similar models used in the cited papers and the authors' previous publications. Specifically: brain-wide imaging (PMID: 35379803, 30692687), PHP.eB AAV validation (PMID: 34139149), Fos+ cell histological analyses (PMID: 35379803, 39549697, 30692687), c-Fos IHC (PMID: 34139149, 39506118), in vitro Fos screening assay (PMID: 32187527), fiber photometry (PMID: 38547869, 38514778), single-cell transcriptomics (PMID: 38326616), RNAscope (PMID: 31031006), behavioral tests (PMID: 35379803, 30692687, 39549697), and ex vivo astrocyte Ca^{2+} imaging (PMID: 34139149, 31031006).
Data exclusions	Tissue samples that yielded insufficient cell counts (<thirty cells) during scRNA-seq quality control were excluded from the study.
Replication	To verify the reproducibility of the experimental findings, all data collection was done in multiple batches, at least twice independently as stated in figure legends. All attempts at replication were successful.
Randomization	All samples were randomly allocated into treatment groups.
Blinding	Investigators were blinded to group allocation during data acquisition for imaging, sequencing, and in vivo recordings. For behavioral experiments, blinding was implemented by using numerical mouse IDs, and whenever possible, data collection and analysis were carried out by different individuals. For in vitro experiments, blinding was not performed because the experimenters needed to know the conditions of each well to conduct the assays appropriately.

Reporting for specific materials, systems and methods

We require information from authors about some types of materials, experimental systems and methods used in many studies. Here, indicate whether each material, system or method listed is relevant to your study. If you are not sure if a list item applies to your research, read the appropriate section before selecting a response.

Materials & experimental systems

n/a	Involved in the study
☐	☒ Antibodies
☐	☒ Eukaryotic cell lines
☒	☐ Palaeontology and archaeology
☐	☒ Animals and other organisms
☒	☐ Clinical data
☒	☐ Dual use research of concern
☒	☐ Plants

Methods

n/a	Involved in the study
☒	☐ ChIP-seq
☐	☒ Flow cytometry
☒	☐ MRI-based neuroimaging

Antibodies

Antibodies used

Primary antibodies:
Rat anti-GFAP, Thermo Fisher Scientific, 13-0300, RRID: AB_2532994, 1:500 for IHC
Mouse anti-S100β, Sigma-Aldrich, S2532, RRID: AB_477499, 1:500 for IHC
Mouse anti-RFP, MBL, M155-3, RRID: AB_1278880, 1:500 for IHC
Rabbit anti-RFP, Rockland, 600-401-379, RRID: AB_2209751, 1:500 for IHC
Rabbit anti-c-Fos, Synaptic Systems, 226003, RRID: AB_2231974, 1:5000 for IHC, 1:1000 for ICC
Mouse anti-NET, Mab Technologies, NET05-2, RRID: AB_2571639, 1:2000 for IHC
Goat-Sox9 (1:100, R&D systems, AF3075), 1:100 for IHC
Mouse anti-c-Fos, Santa Cruz, sc-166940, RRID: AB_10609634, 1:50 for ICC
Rabbit anti-Iba1, Fujifilm, 019-19741, RRID: AB_839504, 1:1000 for ICC
Chicken anti-Tuj1, Novus Biologicals, NB100-1612, RRID: AB_10000548, 1:1000 for ICC
Mouse anti-Olig2, Millipore, MABN50, RRID: AB_10807410, 1:100 for ICC
Rabbit anti-mGluR3, Abcam, ab166608, RRID: AB_2833092, 1:500 for ICC
CD11b-BV786 (M1/70, Lot 4317109, BD Bioscience), 1:200 for FACS
CD45-APC-Cy7 (30-F11, Lot B442084, BioLegend), 1:200 for FACS
O1-eFluor660 (50-6506-82, Lot 2848347, eBioscience), 1:200 for FACS
ASCA2-PE-Cy7 (130-116-246, Lot, 5231104462, Miltenyi Biotec), 1:200 for FACS

Secondary antibodies (1:1000 dilution):
Alexa Fluor 488 donkey anti-rat, Abcam, ab150153, RRID: AB_2737355
Alexa Fluor 488 donkey anti-chicken, ThermoFisher, A78948, RRID: AB_2921070
Alexa Fluor 568 donkey anti-mouse, Abcam, ab175700, RRID: AB_3083693
Alexa Fluor 568 donkey anti-goat, Abcam, ab150129, RRID: AB_2687506
Alexa Fluor 568 donkey anti-rabbit, Abcam, ab175470, RRID: AB_2783823
Alexa Fluor 647 donkey anti-rabbit, Abcam, ab150067, RRID: AB_2894821
Alexa Fluor 647 donkey anti-mouse, Abcam, ab150111, RRID: AB_2890625
Alexa Fluor 647 donkey anti-chicken, Jackson ImmunoResearch, 703-605-155, RRID: AB_234039

Validation

All antibodies were either validated by the manufacturers for the specified species and applications or previously confirmed in the literature. Optimal dilutions were determined empirically on mouse brain tissues, and secondary-antibody-only controls confirmed specificity (no signal without primary). Details for each antibody are as follows:
Rat anti-GFAP (Thermo Fisher Scientific #13-0300, clone 2.2B10, RRID: AB_2532994): Monoclonal IgG2a antibody raised in rat against enriched bovine GFAP filaments. The immunogen was enriched bovine glial filaments. The manufacturer's documentation states that it reacts with mouse, rat, human, and bovine GFAP and is validated for immunohistochemistry and related applications.(https://www.biocompare.com/9776-Antibodies/7012889-GFAP-Glial-Fibrillary-Acid-Protein-Monoclonal-Antibody-2-2B10/). Previously used in PMID: 38302444.
Mouse anti-S100β (Sigma-Aldrich #S2532, clone SH-B1, RRID: AB_477499): Mouse IgG1 monoclonal antibody (clone SH-B1) against S100β. The immunogen was S100β purified from bovine brain. The manufacturer's documentation states that the antibody has been validated for immunohistochemistry and other assays, with broad species reactivity (reacts with human, rat, bovine, pig, goat, etc.) (https://www.sigmaaldrich.com/US/en/product/sigma/s2532?srsltid=AfmBOorJCzKHm_kMMaejg31phReliQI0VB2jbal0Tfv-qbF-cYqt5ahV) . Previously used in PMID: 34139149.
Mouse anti-RFP (MBL International M155-3, clone 8D6, RRID: AB_1278880): Mouse IgG1 monoclonal antibody (clone 8D6) against red fluorescent protein (RFP). The manufacturer's documentation states that the antibody has been validated for immunocytochemistry and immunohistochemistry (https://www.mblbio.com/bio/g/dtl/A/index.html?pcd=M155-3). Previously used in PMID: 37323585.
Rabbit anti-RFP (Rockland #600-401-379, polyclonal, RRID: AB_2209751): Rabbit polyclonal antibody affinity-purified against full-length Discosoma RFP. The immunogen was a recombinant RFP (234 amino acids from Discosoma sp.) The manufacturer's documentation states that the antiserum was immunoaffinity-purified and extensively adsorbed to remove cross-reactivity with mammalian proteins (https://www.rockland.com/categories/primary-antibodies/rfp-antibody-pre-adsorbed-600-401-379/?srsltid=AfmBOooSy5itbhp4CoqwicBqO7rJ2R_FvAMkOQWn17_MoyAj0YIu6SFm). Previously used in PMID: 34139149.
Rabbit anti-c-Fos (Synaptic Systems #226 003, polyclonal, RRID: AB_2231974): Rabbit polyclonal antibody against c-Fos. Immunogen was a synthetic peptide corresponding to amino acids 2–17 of rat c-Fos (UniProt P12841). The manufacturer's documentation states that the antibody has been validated it is affinity-purified and validated for immunohistochemistry on rodent brain tissue (https://fnkprddata.blob.core.windows.net/domestic/data/datasheet/SS2/226003.pdf). Previously used in PMID: 34139149.
Mouse anti-NET (Norepinephrine Transporter; Mab Technologies #NET05-2, clone 2-3B2, RRID: AB_2571639): Mouse IgG2b

monoclonal antibody (clone 2-3B2) raised against a peptide from the mouse norepinephrine transporter (NET) .The manufacturer's documentation states that it specifically recognizes mouse and rat NET on neuronal membranes but does not bind human NET (species specificity confirmed by sequence homology) (https://mabtechnologies.com/categories/product/7-norepinephrine-transporter-mouse-net05-2). Previously used in PMID: 29133432).
Goat anti-Sox9 (R&D Systems AF3075, polyclonal): Goat polyclonal IgG antibody against Sox9. Immunogen was recombinant human SOX9 (Met1–Lys151) expressed in E. coli. The manufacturer's documentation states that the antibody has been validated antigen affinity purification in immunocytochemistry and Western blot (primarily on human samples) (https://www.rndsystems.com/products/human-sox9-antibody_af3075). Previously used in PMID: 28336567.
Mouse anti-c-Fos (Santa Cruz Biotechnology sc-166940, clone E-8, RRID: AB_10609634): Mouse monoclonal IgG1 κ antibody (clone E-8) against c-Fos. The epitope recognized is mapped to amino acids 128–152 of human c-Fos. The manufacturer's documentation states that this clone is validated for immunofluorescence and immunohistochemistry (paraffin) in mouse, rat, human, and even zebrafish tissues (https://www.scbt.com/p/c-fos-antibody-e-8?srsltid=AfmBOoq6tOL6gXYJG5QLW-dcHYBvgg9iCpBg6OakoaDJTwh0HV3lwoQ9). Previously used in PMID: 37229433.
Rabbit anti-Iba1 (FUJIFILM Wako #019-19741, polyclonal, RRID: AB_839504): Rabbit polyclonal antibody against Iba1 (microglial calcium-binding protein). The immunogen is a synthetic peptide corresponding to the C-terminal amino acids of Iba1, a region conserved in mouse, rat, and human. The antibody was affinity-purified and is recommended for immunocytochemistry/immunohistochemistry (but not Western blot) by the manufacturer (https://labchem-wako.fujifilm.com/us/product/detail/W01W0101-1974.html). Previously used in PMID: 33414461.
Chicken anti-βIII Tubulin (TuJ1) (Novus Biologicals NB100-1612, polyclonal, RRID: AB_10000548): Chicken polyclonal IgY antibody against neuron-specific class III β-tubulin (TuJ1 antigen). This antibody was generated by immunizing chickens with synthetic peptides from human TUBB3, and the IgY was affinity-purified. The manufacturer's documentation states that the antibody has been validated for immunofluorescence and immunohistochemistry, and is widely used as a pan-neuronal marker. (https://www.novusbio.com/products/beta-iii-tubulin-antibody_nb100-1612?srsltid=AfmBOopjF61F7ST1-ktgtvSZnbk8thSNz3TsNcuO5_haFp-BGAt_Oczo). Previously used in PMID: 38843836.
Mouse anti-Olig2 (Millipore Sigma MABN50, clone 211F1.1, RRID: AB_10807410): Mouse monoclonal IgG2a antibody (clone 211F1.1) against Olig2 (oligodendrocyte lineage transcription factor). The immunogen was a recombinant human Olig2 protein. The manufacturer's documentation states that the antibody has been validated in Western blot, immunocytochemistry, and immunohistochemistry; it reacts with Olig2 in mouse, rat, and human samples (https://www.merckmillipore.com/INTL/en/product/Anti-Olig2-Antibody-clone-211F1.1,MM_NF-MABN50). Previously used in PMID: 17428828.
Rabbit anti-mGluR3 (Abcam ab166608, clone EPR9009(2), RRID: AB_2833092): Rabbit recombinant monoclonal antibody against mGluR3. The immunogen was a proprietary synthetic peptide from human mGluR3 (GRM3). The manufacturer's documentation states that this clone is validated for ICC/IF and flow cytometry and recognizes mGluR3 in human, mouse, and rat samples (https://www.labome.com/knockout-validated-antibodies/mGlu3-antibody-knockout-validation-Abcam-ab166608.html). Its specificity was further confirmed by knockout validation (showing loss of staining in Grm3–/– tissues). In our hands, this antibody labeled mGluR3 in transfected cell cultures (consistent with manufacturer's validation in HEK293 cells and brain tissue).
CD11b–BV786 (BD Biosciences, clone M1/70): A rat monoclonal IgG2b κ antibody (clone M1/70) against CD11b (integrin α_M) conjugated to Brilliant Violet 786. The manufacturer's documentation states that this clone is a well-characterized antibody that binds mouse CD11b (with demonstrated cross-reactivity to human CD11b) (https://www.bdbiosciences.com/en-us/products/reagents/flow-cytometry-reagents/research-reagents/single-color-antibodies-ruo/bv786-rat-anti-cd11b-integrin-m.569504). Previously used in PMID: 30760929.
CD45–APC-Cy7 (BioLegend, clone 30-F11): A rat monoclonal IgG2b κ antibody (clone 30-F11) against pan-CD45, conjugated to APC/Cy7. Clone 30-F11 recognizes all isoforms of mouse CD45 (including both CD45.1 and CD45.2 alleles). The manufacturer's documentation states that the antibody has been validated for flow cytometry (BioLegend reports routine QC on mouse leukocytes) and does not distinguish between leukocyte subsets since it binds a common epitope on CD45 (https://www.biolegend.com/de-at/products/apc-cyanine7-anti-mouse-cd45-antibody-2530). Previously used in PMID: 30760929.
Oligodendrocyte Marker O1–eFluor660 (Invitrogen eBioscience, clone O1): A mouse IgM monoclonal antibody (clone O1) against galactocerebroside and sulfated lipids on late oligodendrocyte progenitors, conjugated to eFluor™ 660 (equivalent to Alexa 647). The manufacturer's documentation states that this antibody (originally O1 from Sommer and Schachner's work) reacts with oligodendrocytes of multiple species (mouse, rat, human, chicken) because it targets a conserved lipid antigen. (https://www.thermofisher.com/antibody/product/Oligodendrocyte-Marker-O1-Antibody-clone-O1-Monoclonal/50-6506-82). Previously used in PMID: 39009472.
ACSA-2–PE-Cy7 (Miltenyi Biotec #130-116-246, clone REA969): A recombinant human IgG1 monoclonal antibody (REAfinity clone REA969) specific for mouse Astrocyte Cell Surface Antigen-2 (ACSA-2), conjugated to a PE/Cy7-equivalent fluorochrome (PE–Vio770). The manufacturer's documentation states that the antibody has been validated for flow cytometry as a pan-astrocyte marker in mice (https://www.labome.com/product/Miltenyi-Biotec/130-116-246.html). Previously used in PMID: 37845031.
Mouse IGFBP-2 Antibody (R&D Systems #MAB797): Mouse recombinant monoclonal antibody against IGFBP-2. The manufacturer's documentation states that this clone is Measured by its ability to neutralize IGFBP-2 inhibition of IGF-II-dependent proliferation in the MCF-7 human breast cancer cell line (https://www.rndsystems.com/products/mouse-igfbp-2-antibody-150101_mab797). Previously used in PMID: 36042312.
Rat IgG control antibody (R&D Systems # 6-001-F): Polyclonal Rat IgG. The manufacturer's documentation states that it offers controls for flow cytometry, immunohistochemistry, and Western blotting.
(https://www.rndsystems.com/products/mouse-igfbp-2-antibody-150101_mab797). Previously used in PMID: 36042312.

Eukaryotic cell lines

Policy information about cell lines and Sex and Gender in Research

Cell line source(s)	AAVpro 293T cells (Clontech, 632273).
Authentication	The cells were authenticated by the supplier using the STR-based method.
Mycoplasma contamination	The cell line tested negative for mycoplasma contamination.
Commonly misidentified lines (See ICLAC register)	No misidentified cell lines were used.

Animals and other research organisms

Policy information about studies involving animals; ARRIVE guidelines recommended for reporting animal research, and Sex and Gender in Research

Laboratory animals	Young adult (8-12 weeks old) wild-type C57BL/6NCrSlc mice obtained from the Japan SLC, Adra1a flox mice obtained from the RIKEN (RBRC11837) and Adrb1 flox mice (Accession Number: CDB1068K generated by RIKEN Center for Biosystems Dynamics Research https://large.riken.jp/distribution/mutant-list.html) and Fos2A-iCre/ERT2 (TRAP2, JAX Stock 030323) mice obtained from the Jackson Laboratory were used for in vivo experiments. TRAP2 mice were bred and maintained in our animal facility, backcrossed with C57BL/6NCrSlc at least 8 times before use for experiments. Wild-type C57BL/6NCrSlc female mice were obtained from the Japan SLC to obtain postnatal day 0-1 (P0-1) pups. Experiments were done using both male and female mice. Number of mice used in each experiment is listed in the main text and/or figure legends accordingly. Mice were housed in groups of two to five in a temperature- and humidity-controlled room (23 ± 3 °C, 45 ± 5% humidity) under a 12-hour (h) light–dark cycle (lights on from 7 AM to 7 PM) and given ad libitum access to water and laboratory mouse diet at all times. After fiber implantation, the mice were single housed.
Wild animals	The study did not involve wild animals.
Reporting on sex	Animals of both sexes were included in the study and sex was not considered in random assignments to experimental groups, particularly due to the difficulty of distinguishing sexes in neonatal mice for in vitro experiments.
Field-collected samples	The study did not use field-collected samples.
Ethics oversight	All experiments were approved by the RIKEN Animal Care and Use Committee.

Note that full information on the approval of the study protocol must also be provided in the manuscript.

Plants

Seed stocks	n/a
Novel plant genotypes	n/a
Authentication	n/a

Flow Cytometry

Plots

Confirm that:

☒ The axis labels state the marker and fluorochrome used (e.g. CD4-FITC).

☒ The axis scales are clearly visible. Include numbers along axes only for bottom left plot of group (a 'group' is an analysis of identical markers).

☒ All plots are contour plots with outliers or pseudocolor plots.

☒ A numerical value for number of cells or percentage (with statistics) is provided.

Methodology

Sample preparation	Mice were anesthetized with isoflurane 90 min after the initiation of behavioral paradigms and transcardially perfused with 1x PBS (30 mL for 2 min). The amygdala was freshly dissected out using a Brain Matrix (Ted Pella, 15067) and was placed into cold Hanks' balanced salt solution (HBSS) (14025092, Gibco) containing 10 mM HEPES (17514-15, Naclai Tesque), 0.54 % glucose (16806-25, Naclai Tesque), 5.0 µg/mL Actinomycin D (A1410, Sigma-Aldrich), 10 µM Triptolide (T3652, Sigma-Aldrich) and 27.1 µg/mL Anisomycin (A9789, Sigma-Aldrich), as previously described (Yamasaki et al., Commun Biol 2024; Marsh et al., Nat Neurosci 2022). Then, the tissue block was incubated for 15 min at 37°C in a 3 mL enzyme solution (1x HBSS, 10 mM HEPES, 0.54 % glucose, 0.5 mM EDTA (15575020, Invitrogen), 1.0 mM L-Cys (10309-41, Naclai Tesque), 5.0 µg/mL Actinomycin D, 10 µM Triptolide and 27.1 µg/mL Anisomycin) containing 20 U of papain per mouse (LS003127, Worthington Biochemical). The tissue was then homogenized by passing through a 18G needle and was further incubated for 15 min at 37 °C. Following homogenization using a 20G needle, the resulting cell suspension was centrifuged at 500 x g for 5 min at 4 °C. The homogenate was separated by gradient centrifugation with 30% Percoll (P1644, Sigma-Aldrich) in 1x PBS at 500 x g for 25 min at 4°C (no brake). The pellet containing astrocytes at the bottom of the tube was then collected and washed once with PBS containing 2 % FBS and 10 mM EDTA before staining. Fc receptors were blocked with Fc block (2.4G2, BD Bioscience) for 10 min at 4°C before incubation with primary antibodies. Cells were stained with antibodies directed against CD11b-BV786 (M1/70, BD Bioscience), CD45-APC-Cy7 (30-F11, BioLegend), O1-eFluor660 (50-6506-82, eBioscience) and

	ASCA2-PE-Cy7 (130-116-246, Miltenyi Biotec) for 40 min at 4°C. Additionally, cells were treated with hashtag antibodies to label source samples (TotalSeqB0301-B0310, B0312 and B0314, BioLegend).
Instrument	Cells were sorted using a CytoFlex SRT (Beckman Coulter)
Software	Data were acqired with CytExpert software (Beckman Coulter). Post aquisition analysis was perfomed using FlowJo software, version 10.9.0.
Cell population abundance	The cell population abundances are provided in the plots depicting the representative gating strategies of each experiment.
Gating strategy	In all experiments, small debris was removed with the preliminary FSC/SSC gate. Single cells were obtained by doublet exclusion. Further, gating strategies for the respective experiments are provided in the figures.

☒ Tick this box to confirm that a figure exemplifying the gating strategy is provided in the Supplementary Information.
