# Supplementary material for: The astrocytic ensemble acts as a multiday trace to stabilize memory
Source: Nature. 2025 Oct 15;648(8092):146–56. doi: 10.1038/s41586-025-09619-2 (PMC12675280; doi:10.1038/s41586-025-09619-2)
Supplement: Supplementary file 5 — AAV constructs and Addgene IDs. [file 41586_2025_9619_MOESM5_ESM.docx]

| **AAV construct** | **Addgene ID** | **Titer** |
| --- | --- | --- |
| *AAV-PHP.eB *GfaABC_1_D*-DIO-mNeonGreen-mScarlet-I-WPRE | Addgene 223668 | 2.5 x 10^12^ GCs in 50 µL |
| *AAV-PHP.eB *GfaABC_1_D*-DIO-mNeonGreen-WPRE | Addgene 223669 | 2.5 x 10^12^ GCs in 50 µL |
| *AAV2/5 *GfaABC_1_D*-DIO-mNeonGreen-WPRE | Addgene 223669 | 3.0 x 10^12^ GCs/mL |
| *AAV2/DJ8-*RAM*-d2TTA::TRE-hM4Di-mCherry-WPREpA | Addgene 223670 | 3.0 x 10^13^ GCs/mL |
| *AAV2/5 *GfaABC_1_D*-mGrm3-WPRE | Addgene 224236 | 8.0 x 10^13^ GCs/mL |
| *AAV2/5 *GfaABC_1_D*-flag-mAdrb1-WPRE | Addgene 223671 | 3.0 x 10^12^ GCs/mL |
| *AAV2/5 *GfaABC_1_D*-flag-mAdrb1-mNeonGreen-WPRE | Addgene 223672 | 3.0 x 10^12^ GCs/mL |
| *AAV2/5 *hSyn1*-mCherry-CAAX | Addgene 223674 | 3.0 x 10^12^ GCs/mL |
| *AAV2/5 *hSyn1*-DIO-mCherry-CAAX | Addgene 223673 | 3.0 x 10^12^ GCs/mL |
| AAV2/9 rTH-PI-Cre | Addgene 107788 | 3.0 x 10^12^ GCs/mL |
| AAV2/8 *hSyn1*-DIO-hM3Dq-mCherry | Addgene 44361 | 1.0 x 10^13^ GCs/mL |
| AAV2/8 *hSyn1*-DIO-hM4Di-mCherry | Addgene 44362 | 1.0 x 10^13^ GCs/mL |
| AAV2/8 *hSyn1*-DIO-mCherry | Addgene 50459 | 3.0 x 10^12^ GCs/mL |
| AAV2/5 *GfaABC_1_D*-hM3Dq-mCherry | Addgene 92284 | 3.0 x 10^12^ GCs/mL |
| AAV2/DJ8 *Ef1a-*hM3Dq-mCherry | N/A | 4.8 x 10^11^ GCs/mL |
| AAV2/DJ8 *Ef1a-*hM4Di-mCherry | N/A | 4.0 x 10^12^ GCs/mL |
| AAV2/9- *hSyn1*-GRAB_NE2h_-WPRE | BrainVTA PT-5262 | 2.7 x 10^12^ GCs/mL |
| AAV2/1 *GfaABC_1_D* cAMPinG1-NE | N/A | 1.3 x 10^13^ GCs/mL |
| AAV2/1 *GfaABC_1_D*-RCaMP3 | N/A | 1.5 x 10^13^ GCs/mL |
| AAV2/5 *GfaABC1D*-cyto**-**GCaMP6f | Addgene 52925 | 1.1 x 10^13^ GCs/mL |
| AAV2/5 *GfaABC1D*-iβARK-p2A-mCherry | Addgene 117691 | 1.3 x 10^12^ GCs/mL |
| AAV2/5 *GfaABC1D*-iβARK(D110A)-p2A-mCherry | Addgene 117692 | 1.3 x 10^12^ GCs/mL |
| *AAV2/5 *GfaABC1D*-Lck-mCherry-iβARK | Addgene 241241 | 1.3 x 10^12^ GCs/mL |
| *AAV2/5 *GfaABC1D*-Lck-mCherry-iβARK(D110A) | Addgene 241242 | 1.1 x 10^13^ GCs/mL |
| *AAV2/5 *GfaABC1D*-DIO-Lck-mCherry-iβARK-4x6T | Addgene 241243 | 3.0 x 10^13^ GCs/mL |
| *AAV2/5 *GfaABC1D*-DIO-Lck-mCherry-iβARK(D110A)-4x6T | Addgene 241244 | 3.0 x 10^13^ GCs/mL |
| *AAV2/5 *Fos*-Flpo^ERT2^ | Addgene 241239 | 3.0 x 10^12^ GCs/mL |
| *AAV2/5 *GfaABC1D*-fDIO-mNeonGreen | Addgene 241240 | 1.0 x 10^12^ GCs/mL |
| AAV2/5 *GfaABC1D*-Cre-4x6T | Addgene 196410 | 1.2 x 10^13^ GCs/mL |

**Supplementary Table 4**. AAV constructs used in this study (* 15 new AAVs generated in this study).
